# Treatment of nail psoriasis with intralesional methotrexate: report of four cases demonstrating an effective and safe approach with lower doses^☆^

**DOI:** 10.1016/j.abd.2024.05.001

**Published:** 2024-10-30

**Authors:** Angélica Seidel, Marcelo Rigatti, Débora Cadore de Farias, Ana Paula Bald, José Ricardo Grams Schmitz

**Affiliations:** Hospital Universitário Polydoro Ernani de São Thiago, Universidade Federal de Santa Catarina, Florianópolis, SC, Brazil

Dear Editor,

The prevalence of nail psoriasis (NP) ranges from 10% to 82% among patients with psoriasis, with a lifetime incidence of 80% to 90%. It is considered one of the most difficult regions to treat, with 5% to 10% of cases manifesting in the absence of cutaneous symptoms. It may be associated with pain, inflammation with paronychia, aesthetic concerns, and impaired finger function, which significantly impacts the quality of life of individuals.^1^, ^2^

Topical and injectable therapies are recommended for disease affecting a few nails (<3) and involve local injections of steroids, such as triamcinolone acetonide (TAC), methotrexate, and also cyclosporine. Studies indicate TAC as the most investigated therapy. Intramatrix injection of methotrexate showed the greatest benefit with minimal side effects and results comparable to TAC injection. Cyclosporine was the least effective medication and had the most side effects.^1^, ^3^

Due to the reported efficacy of intralesional MTX in recent studies, it can be considered among the first-line therapies, especially in cases of nail matrix involvement, and also when efficacy with minimal side effects is prioritized. Studies indicate a dose of 2.5 mg per session (0.1 mL of a 25 mg/mL solution) in the nail bed, with local pain being the main reported side effect.^4^, ^5^, ^6^

Systemic treatment, including biologicals, is recommended in resistant cases, when three or more nails are affected, in cases of extensive involvement of the skin and joints, or/and a significant impact on patient quality of life.^7^, ^8^, ^9^

Herein, the authors report a series of four cases treated in their service, using intramatrix methotrexate at a reduced dose, with details of the technique used and satisfactory results.

## Patient 1

A 53-year-old female patient, previously diagnosed with fibromyalgia, dyslipidemia, and diabetes, was taking amitriptyline, simvastatin and metformin. She had had a diagnosis of psoriasis vulgaris with nail involvement for more than ten years. She was receiving subcutaneous methotrexate 25 mg/week and folic acid 5 mg/week, and the skin condition was under control. She was also receiving topical clobetasol 8% in nail polish twice a week. She underwent intramatrix infiltration of methotrexate in both thumbs and hallux. The technique consisted of aspirating 12.5 mg of the medication (0.5 mL) with 0.5 mL of saline solution, of which 0.05 mL (0.625 mg of methotrexate) was applied divided into two injections bilaterally (0.025 mL at each site located 3 mm from the proximal digital fold ‒ angle of intersection of the nail with the digital fold) on each thumb and hallux. The total dose for the patient was 2.5 mg; a 100 IU (1 mL) BD syringe was used.

## Patient 2

A 68-year-old female hypertensive patient, with heart disease, dyslipidemia, and hypothyroidism, had had a diagnosis of NP for more than one year. Previous treatments included topical corticosteroids and oral biotin. She underwent three sessions of intramatrix methotrexate, at monthly intervals, with the application of 0.625 mg (0.05 mL of the solution) in all fingernails, according to the technique described above. The total dose per session was 6.25 mg.

## Patient 3

A 78-year-old male hypertensive patient, with heart disease, benign prostatic hyperplasia, labyrinthitis, diverticulitis, and urticaria, had had a diagnosis of NP and lichen planus for more than two years. Previous treatments included topical corticosteroids, urea, erythromycin, terbinafine, oral acitretin (25 mg/day), and oral zinc. He underwent the same protocol as patient number 2.

## Patient 4

A 67-year-old female patient with depression and frontal fibrosing alopecia had had a diagnosis of NP for five months. Previous treatments included urea and corticosteroids. He underwent the same protocol as patient number 2.

Table 1 present the clinical cases of the present study (Table 1).

**Table 1:** Clinical cases.

Case	Gender	Age	Nail Psoriasis Evolution Time	Previous treatments	Nail Changes – Before and After	Number of sessions	Dose per Nail Fold	Number of Treated Finger/Toes	Patient Satisfaction Level	Interval between the sessions
1	Female	53 years old	>10 years	Subcutaneous methotrexate, topical clobetasol		1	1.25 mg (0.05 mL of the solution)	Bilateral thumbs + hallux	High	N/A
2	Female	68 years old	>1 year	Topical corticosteroids, oral biotin		3	0.625 mg (0.05 mL of the solution)	All fingernails	High	Monthly
3	Male	78 years old	>2 years	Topical corticosteroid, urea, erythromycin, terbinafine, oral acitretin, oral zinc		3	0.625 mg (0.05 mL of the solution)	All fingernails	High	Monthly
4	Female	67 years old	5 months	Urea, high potency topical corticosteroid		3	0.625 mg (0.05 mL of the solution)	All fingernails	High	Monthly

After reviewing the literature and the case series presented above, it is possible to observe that intramatrix therapy is a safe, cost-effective and sucessfull therapeutic modality in the management of NP. Nevertheless, to date, there is no literature that demonstrates protocols with doses and forms of administration of the medication. By describing these cases, the authors believe they have filled this gap. Using the described technique, with reduced doses, in addition to promoting greater patient comfort, one can reserve higher concentrations for patients who do not respond to initial sessions. However, for it to be considered a promising therapeutic approach, the authors emphasize the need for additional studies.

## Financial support

None declared

## Authors' contributions

Angélica Seidel: Approval of the final version of the manuscript; design and planning of the study; drafting and editing of the manuscript; critical review of the literature.

Marcelo Rigatti: Effective participation in research orientation; intellectual participation in the propaedeutic and/or therapeutic conduct of the studied cases; critical review of the manuscript.

Débora Cadore de Farias: Effective participation in research orientation; intellectual participation in the propaedeutic and/or therapeutic conduct of the studied cases; critical review of the manuscript.

Ana Paula Bald: Drafting and editing of the manuscript; critical review of the literature.

José Ricardo Grams Schmitz: Drafting and editing of the manuscript; critical review of the literature.

## Conflicts of interest

None declared.
